# A V-Shaped Microcantilever Sensor Based on a Gap Method for Real-Time Detection of *E. coli* Bacteria

**DOI:** 10.3390/bios12040194

**Published:** 2022-03-25

**Authors:** Jino Fathy, Yongjun Lai

**Affiliations:** Department of Mechanical and Materials Engineering, Queen’s University, Kingston, ON K7L 3N6, Canada; 17jf14@queensu.ca

**Keywords:** microcantilever, sensor, dielectrophoresis, *E. coli* bacteria

## Abstract

This paper presents a dynamic-mode microcantilever sensor based on a gap method. The sensor has a V-shaped microcantilever and a fixed structure at a distance of 2 µm from its free end. The microcantilever is excited by applying an ac electric potential (3 V_p_) to its piezoelectric pads and vibrates at its fundamental resonant frequency. An independent ac electric potential (200 kHz, 15 V_pp_) is applied to the fixed structure. This creates a non-uniform electric field with its maxima at the gap and exerts a dielectrophoresis (DEP) force. The DEP force attracts and adsorbs the *E. coli* bacteria to the cantilever edge at the gap. The binding of the bacteria to the cantilever creates a shift in the resonant frequency of the microcantilever sensor, which is detected by a laser vibrometer. The real-time detection of *E. coli* bacteria samples, diluted in distilled water, was performed for concentrations of 10^5^–10^3^ cells/mL and the real-time frequency shifts were −2264.3 to −755 Hz in 4 min, respectively. The tests were expanded to study the effect of the electric potential amplitude (10, 12, 15 V_pp_) and higher frequency shifts were observed for higher amplitudes.

## 1. Introduction

The detection of bacteria in real time and low concentration is in high demand for prevention, treatment, and drug monitoring. Most of the current biosensing methods such as agar plates and broth dilution assays are time consuming and require expert personnel to prepare the sample and analyze the data [1]. Enzyme-linked immunosorbent assay (ELISA) and polymerase chain reaction (PCR) are among well-established detection techniques [2,3]. However, they require purification and enrichment of samples prior to the test. Electrochemical impedance spectroscopy (EIS) provides a good performance, but it requires functionalization of the sensor before the test [4].

Recently, some research reported not only the detection of the bacteria but an expansion in the biosensors’ applications. Differentiation between live and dead bacteria is not easy, as their chemical composition is not significantly different. Zhou et al. reported a label-free in situ surface-enhanced Raman Scattering (SERS) mapping process to discriminate between live and dead bacteria [5]. The bacterial viability and effect of antibiotics have been studied in [6,7] by utilizing optical methods. In addition, Etayash et al. reported a microfluidic cantilever to detect bacteria and study the impact of antibiotics on them [8].

Here, we introduce a new microcantilever sensor. Microcantilevers (MCLs) have provided various applications, including humidity sensors [9,10], therapeutic drug monitoring (TDM) [11], simultaneous measurement of the fluid viscosity and density [12], and the detection of molecules, DNAs, pathogens, and cancer cells [13,14,15,16]. Their minute size and low-cost mass-production make them a good potential for point of care (PoC) and label-free sensing with high sensitivity. 

MCLs may detect the analyte in static or dynamic mode. In static mode, binding the analyte to the cantilever changes its surface stress and bends it so the cantilever deflection is measured. In dynamic mode, the sensing method relies on the shift in the resonant frequency of a vibrating cantilever when the analyte binds to it. Dynamic-mode cantilever sensors, which are the focus of this research, have shown higher sensitivity compared to the static mode. Additionally, they are more reliable in quantitative and/or qualitative measurements of bioanalytes [17,18].

Although most dynamic-mode cantilevers sense the analytes by the change in the effective mass (*m_n_*), the change in the stiffness (*k*) also results in a shift (Δ*f*) in its resonant frequency (*f_n_*) [17,19]:

(1) $$\Delta f=\frac{1}{2}f_{n}\left(\frac{\Delta k}{k}-\frac{\Delta m}{m_{n}}\right)$$

where Δ*k* and Δ*m* are the changes in the stiffness and the mass of the cantilever, respectively. However, in most biosensing applications, the mass-change effect outweighs the stiffness-change effect. Accordingly, the sensitivity *σ_n_* of a dynamic-mode cantilever is expressed only based on its mass-change sensitivity [17,20]:

(2) $$\sigma_{n}=2\frac{m_{n}}{f_{n}}$$

To sense the mass change of the cantilever in dynamic mode, several methods have been introduced in the literature. In suspended microchannel resonators (SMRs), the particle suspension flows into a microchannel that is resonating in a vacuum [21,22]. In another method, particles are collected on the cantilever, and then the cantilever dries to perform the measurements in air or a vacuum [23,24,25]. Some researchers amplify the mass change by adding gold nanoparticles to the target bioanalytes [14,26]. It increases the mass of the target particles, resulting in a more sensitive detection. Besides these techniques, it has been shown that utilizing millimeter-sized cantilevers in higher vibrating modes is effective in measuring the cantilever mass change [27,28].

Researchers are constantly pursuing the development of the real-time detection of bioparticles in low concentrations. Electrokinetics phenomena such as dielectrophoresis (DEP) and ac electroosmosis (ACEO) are promising methods to achieve this goal. They have been used to concentrate particles before entering the sensing section. This improves the detection of low concentration samples, because a higher number of particles, compared with the original dilute sample, are available to be detected [29,30,31]. Furthermore, in some works, electrodes have been implemented on the cantilevers or in the sensing chamber to apply DEP and/or ACEO to the particles. The goal is to accelerate the collection of the particles to the sensor surface and secure them on it, which results in real-time sensing [20,32,33,34,35,36].

The present design utilizes the DEP effect to bring *E. coli* bacteria to the desired locations. DEP is a force exerted by a non-uniform electric field on electrically neutral particles [37]:

(3) $$\langle F_{DEP}\rangle =2\pi \varepsilon_{0}\varepsilon_{m}r^{3}Re\left[{\tilde{f}}_{CM}\right]\nabla {\left|E_{rms}\right|}^{2}$$

where *ε*_0_ and *ε_m_* are the vacuum and the suspending medium permittivity, respectively, *r* is the particle radius, and *E_rms_* is the root mean squared electric field strength. $Re\left[{\tilde{f}}_{CM}\right]$ represents the real part of the Clausius–Mossotti (CM) factor, which indicates if the particles move towards the electric field maxima (positive DEP, pDEP) or against it (negative DEP, nDEP) [37].

The reported sensor in this work is a V-shaped MCL that works based on the gap method. In the gap method, a fixed structure (wall) is secured in a few microns distance from the free end of a vibrating cantilever forming a small gap. The cantilever is grounded, and an independent ac electric potential is applied to the wall. This electrical configuration creates a non-uniform electric field, with its maxima at the gap. Thus, the pDEP force attracts and holds the particles to deposit on the cantilever edge at the gap. It is thought that the particles (or a chain of particles depending on the size of the particles and the gap) bridge the free end of the vibrating cantilever and the wall. This creates significant damping to the MCL vibration and reduces its frequency. Most of these particles are released as soon as the DEP electric potential is off which causes the resonant frequency of the cantilever to partially rebound. 

The gap method has shown promising results for sensitive and real-time sensing in the fundamental resonant frequency of the MCL [20,34]. Detection in the first resonating mode is an advantage because higher modes are hard to excite in liquid due to high liquid damping. Noise may outweigh the higher modes and make them hard to detect, as well. Besides, gap-method microcantilever sensors are label-free, reusable, easily configurable, and have simple preparation, cleaning, and reactivation steps.

Here, we use the gap method technique to detect low concentrations of *E. coli* bacteria in distilled water. The gap between the tip of the V-shaped cantilever and the wall is 2 µm, as designed. A laser vibrometer was used to measure the resonant frequency of the cantilever sensor. The experimental results show the frequency shift of the MCL for 10^5^–10^3^ cells/mL in a stagnant liquid.

## 2. Materials and Methods

### 2.1. Design

**Figure 1:**
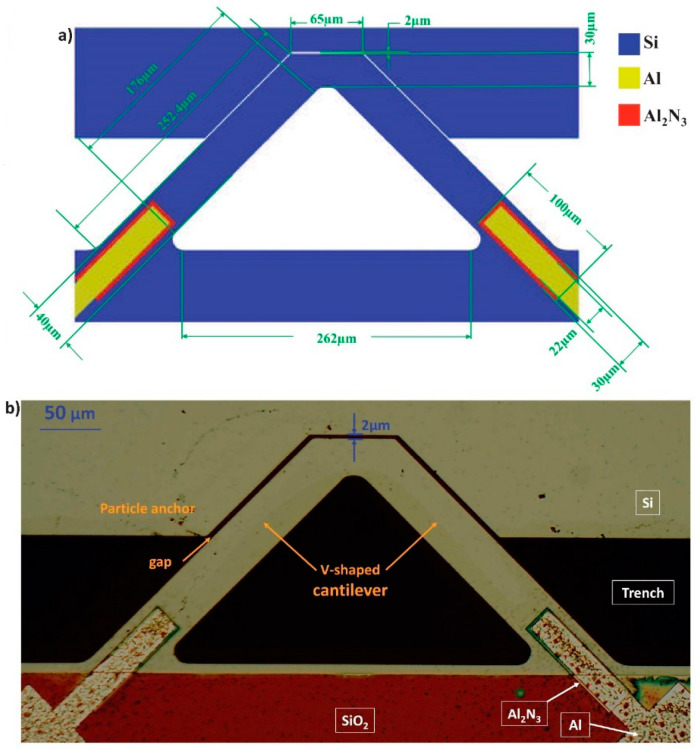
(**a**) Schematic showing the dimensions of the gap-method V-shaped microcantilever. (**b**) A top view image of a fabricated gap method V-shaped cantilever sensor. The materials/layers are indicated in white color and inside boxes. The gap is 2 µm.

The sensor chip was fabricated by the PiezoMUMPs process. It is a micromachining process to fabricate piezoelectric materials on an SOI (Silicon-On-Insulator) chip [38]. The chip has four layers: Si, oxide, metal (Al), and a piezoelectric material (aluminum nitride Al_2_N_3_). The cantilever sensor has a V-shaped structure that is suspended above a trench. There is a fixed structure with a 2 µm distance from the free end of the cantilever as designed. Figure 1a illustrates a schematic of the gap-method V-shaped microcantilever sensor with the dimensions specified. Figure 1b shows a photo of the fabricated cantilever, taken by a 10× microscope. Two electrically connected piezoelectric pads on the anchors drive the cantilever. The piezoelectric strips are sandwiched between the doped silicon on the bottom (grounded) and the aluminum electrode on the top (piezoelectric driving signal, 3 V_p_). The wall is connected to an independent 200 kHz ac signal.

Having a small capture area in the cantilever sensors limits their performance, and their sensitivity reduces as the added mass increases [8,39]. In addition, it has been reported in the previously designed gap-method microcantilevers that they saturate quickly (in about 6 min) [20,34]. Thus, we designed our gap-method microcantilever sensor to have a longer gap, which provides a bigger interaction area to capture more particles and an extended saturation time.

Additionally, it has been shown in [20] that a smaller gap results in a higher frequency shift. In the previously reported gap-method microcantilever sensors, the minimum fabricated and tested gap was 2.7 µm, whereas, in this design, we decreased the gap size to 2 µm (at the orthogonal direction) as designed. 

The fillet shapes at the corners of the microcantilever are implemented to follow the PiezoMUMPs design rules and avoid cracking of the structure during the fabrication process; they have minimal effect on the design performance.

### 2.2. Dynamic Response

**Figure 2:**
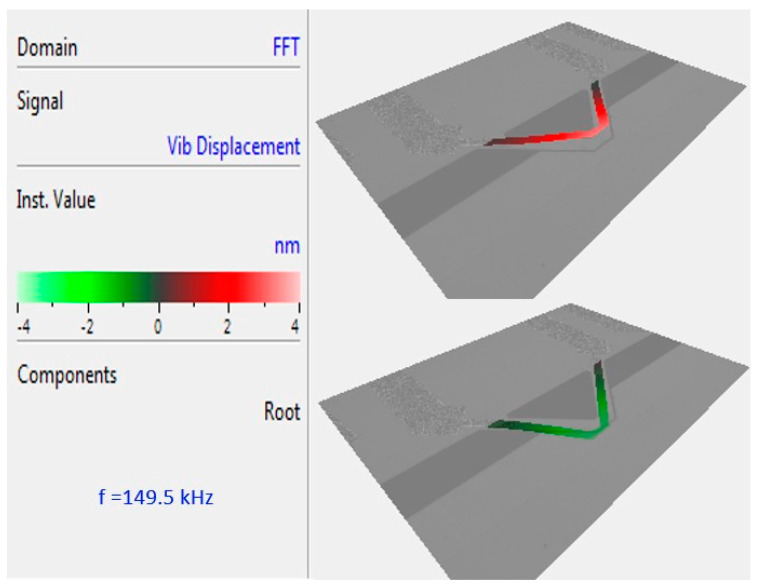
Multiple points scanning of the V-shaped cantilever in distilled water representing its first resonant mode at 149.5 kHz.

Figure 2 shows the mode shape of a fabricated gap method V-shaped cantilever in distilled water collected from a laser vibrometer (MSA-400 Micro System Analyzer, Polytec Inc., Irvine, CA, USA). The microcantilever’s out-of-plane vibration is measured from 50 kHz to 200 kHz and indicates a first mode resonant frequency at 149.5 kHz. The demonstrated mode shape of the resonating cantilever is provided directly by the Polytech software. It represents a map of the measured points all over the cantilever. The quality factor of the cantilever is also measured by the Polytech software, which is 14.4 in an *E. coli* suspension with a concentration of 10^5^ cells/mL.

### 2.3. Sample Preparation

A sample of *E. coli* K-12 in DI water with a concentration of 10^8^ cells/mL was used. The sample was serially diluted by distilled water (150 µL *E. coli* sample with 1350 µL distilled water) to obtain the desired bacteria concentration. In each dilution step and before each test, the samples were mixed on a vortex mixer (Scientific Industries Vortex-Genie 2, Scientific Industries Inc., Bohemia, NY, USA) for 1 min. Then, a 500 µL glass syringe (Hamilton 81220, Hamilton company, Reno, NV, USA) was loaded with the desired sample (distilled water or *E. coli* sample suspension) and was ready to be used for the tests.

### 2.4. Microfluidic Platform

The sample was delivered to the sensor by a customized microfluidic platform. It is made out of two 70 mm × 30 mm × 1.5 mm PMMA plates, tubing, and a PDMS gasket. A laser cutter (Accuris Powersharp 42) was used to cut inlet and outlet ports in the top layer (power 100% and speed 40%) and engrave a 15 mm × 1.5 mm × 0.6 mm (L × W × H) channel in the bottom layer (power 70% and speed 40%) to connect the two ports. Then, the two PMMA pieces were bonded using acetone. Tubing (12.5 cm of SILASTIC 508-003) was connected to the inlet.

On top of the outlet, a thin layer of PDMS (15:1 *w*/*w*) was placed as a gasket. It held the chip in place and created a seal between the microfluidic platform outlet and the chip. A thin sheet of PDMS (Sylgard 184, Sigma-Aldrich Canada Co., Oakville, ON, Canada) was spin-coated (Laurell WS- 400-6NPP/LITE, Laurell Technologies Corporation, North Wales, PA, USA) and cured in an oven (60 °C for 1 h) and then cut with a scalpel. A hole was punched through it by a needle and then aligned over the microfluidic platform outlet port.

### 2.5. Test Setup

**Figure 3:**
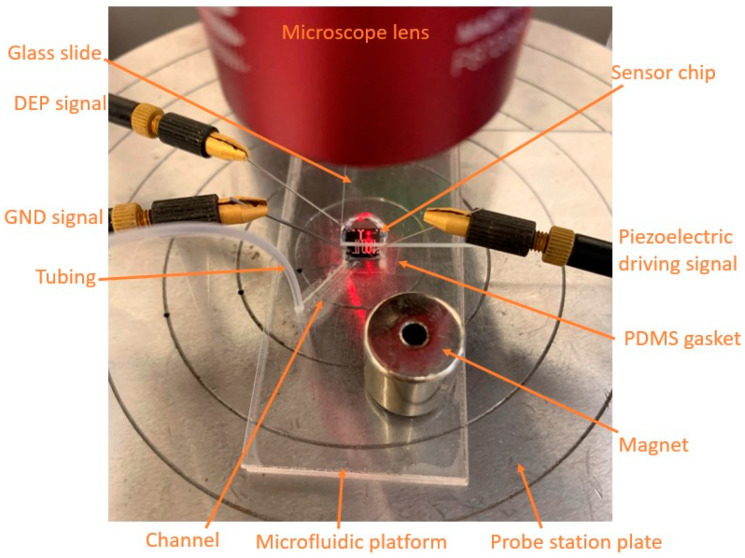
Experimental setup.

The sensor chip was placed on the microfluidic platform and aligned so the trench of the chip was on the outlet hole of the microfluidic platform. Using a tweezer, light pressure was applied on the corners of the chip to create a seal. The microfluidic platform, with the sensor on it, was secured on the plate of a probe station (Polytech MSA-400 Micro System Analyzer, Polytec, Inc. (USA), Irvine, CA, USA) by a magnet. Using a 10× microscope, the three signal probes were lowered onto the electrical pads of the chip: one for grounding the cantilever, one for driving the piezoelectric material, and one for applying the DEP signal to the wall (Figure 3). Then, a glass slide fixed on a probe positioner was slid to cover the sensor with a distance from it. The glass slide was used to flatten the droplet to be able to measure the frequency of the microcantilever by the laser.

A glass syringe was loaded with 150 µL of the liquid (*E. coli* sample or distilled water). Tubing was fitted on the needle of the syringe, and the plunger of the syringe was gently pushed to remove the air in the tubing and fill it with the sample (50 µL is enough to fill the tubing). Then, the other end of the tubing was fitted into the inlet of the microfluidic platform, and the plunger of the syringe was gradually pushed so a 70 µL droplet of liquid immersed the sensor. Then, the microscope was readjusted to see the cantilever inside the liquid. The measurement laser was focused on the center of the tip of the V-shaped microcantilever.

The first mode resonant frequency of the microcantilever was measured for 6 min with 10 s intervals using a code written in VBA. During the first minute, the DEP signal was off then at minute 1 of the test the DEP collection of the bacteria was started and lasted up to minute 5, where the DEP signal was turned off so the resonant frequency of the cantilever rebounds to some extent. The resonant frequency of the cantilever had some small fluctuations. To normalize these fluctuations, the frequencies obtained during the first minute of the test, when the DEP signal had not been activated yet, were averaged, and the frequency shift was calculated in accordance with this average value.

Before and after each test, the sensor chip was washed with distilled water for 15 s and placed on a hot plate (Corning 6795-400D PC-400D) at a temperature of 450 °C for over 10 min to remove any biological components on the sensor. In addition, at the end of each test, the microfluidic platform, the glass slide, the syringe, and the tubing were washed with distilled water.

### 2.6. Control Tests

Two types of control tests were performed. Negative control tests were conducted using distilled water and applying DEP signal in the same pattern as other tests. In the positive control tests, *E. coli* suspension with a concentration of 10^4^ cells/mL was used, and no DEP signal was applied throughout the test.

## 3. Results

**Figure 4:**
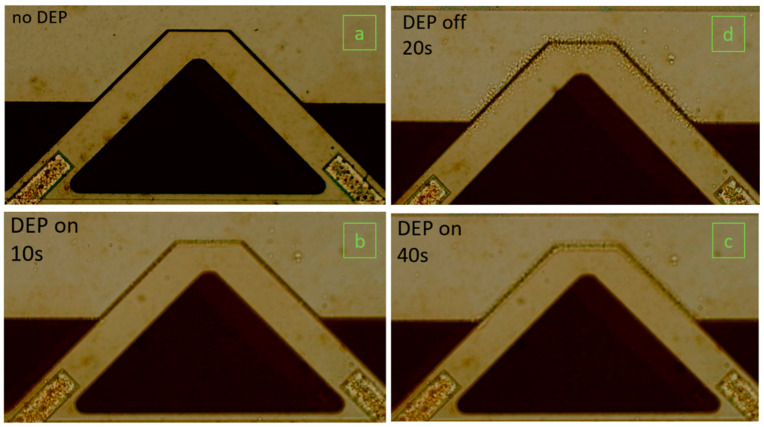
Sequence images of the V-shaped microcantilever immersed in *E. coli* suspension with a concentration of 10^7^ cells/mL when (**a**) the DEP signal has not been activated yet, (**b**) the DEP signal is on for 10 s, (**c**) the DEP signal is on for 40 s, (**d**) the DEP signal is off for 20 s after it was applied for 4 min.

Figure 4 shows a series of images of the gap method V-shaped microcantilever sensor in a 10^7^ cells/mL *E. coli* bacteria suspension. In the beginning, the DEP signal is off, so no particles are observed at the gap (Figure 4a). Then, the DEP signal is on for 10 and 40 s, and the bacteria start moving towards the higher electric field sections at the gap (Figure 4b,c). Finally, the DEP signal is turned off, and the bacteria are released (Figure 4d shows the bacteria release at 20 s after the time the DEP signal is deactivated).

**Figure 5:**
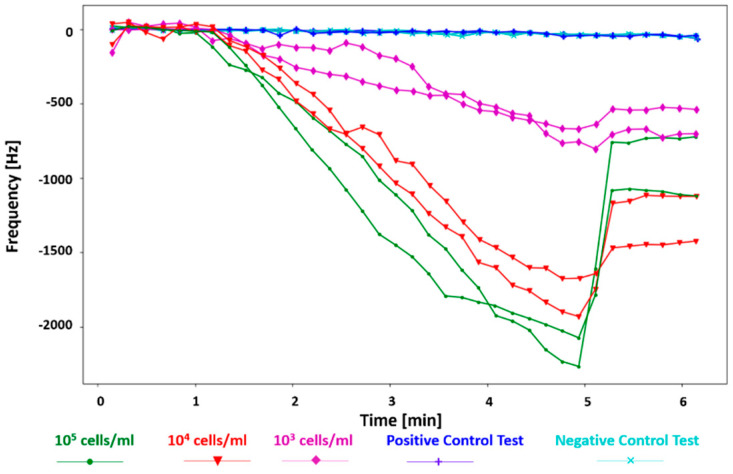
Frequency shifts of the gap-method V-shaped microcantilever sensor for *E. coli* bacteria with 10^5^–10^3^ cells/mL concentrations and the positive and negative control tests.

Figure 5 shows the test results for the *E. coli* samples with 10^5^–10^3^ cells/mL concentrations and the control tests, plotted in Python. For the tests with the *E. coli* samples, the first 1 min shows there is no significant shift in the resonant frequency of the sensor because the DEP signal has not been applied yet. However, as soon as the DEP signal is activated, the bacteria bind to the cantilever edges at the gap, and the shift in the frequency is observed (minutes 1 to 5). Finally, at minute 5 (after the DEP signal was applied for 4 min), the DEP signal is off, so the resonant frequency rebounds to a frequency closer to the main resonant frequency.

A higher frequency shift is observed for higher concentrations of bacteria. The max frequency shifts in 4 min of applying the DEP signal for 10^5^, 10^4^, and 10^3^ cells/mL are −2264.3 Hz, −1929.3 Hz, and −755 Hz, respectively. This is while the frequency shifts for the control tests (with distilled water and *E. coli* 10^4^ cells/mL sample suspension) are negligible, less than −50 Hz at minute 6 (at the end of the test). The signal-to-noise ratio (SNR) of the sensor was calculated by dividing the resonant frequency shift of the sensor right before the DEP signal was turned off by the RMS noise from the positive control test [20]. To consider the worst-case scenario, the lowest frequency shift detected by the sensor, which is the minimum frequency shift observed for 10^3^ cells/mL (−543.5 Hz, not shown here) and the maximum RMS noise from the positive control tests (16.72 Hz) were used. The resulting minimum SNR is 32.5. 

**Figure 6:**
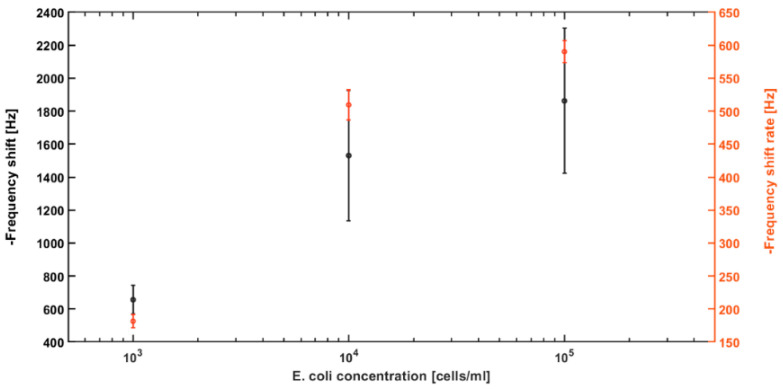
Average frequency shifts and frequency shift rate (n = 3) for different concentrations (10^3^, 10^4^, 10^5^ cells/mL) of *E. coli* bacteria in distilled water. The error bars represent the standard deviation. The right vertical axis (in red color) and the red points and error bars show the frequency shift rate for each concentration in Hz/min.

It is observed that the frequency recovers in various degrees for different concentrations when the DEP signal is deactivated. This is because the loosely bonded bacteria to the cantilever, under the DEP force, are released. In tests with higher bacterial concentrations, more bacteria are attracted and attached to the cantilever; thus, a higher number of them are released when the DEP signal is deactivated, which leads to a larger frequency recovery. Additionally, the rate of the frequency shift for each concentration of the *E. coli* suspension was calculated in Hz/min for comparison. The average frequency shift rates for the gap method V-shaped cantilever sensor for concentrations of 10^5^–10^3^ cells/mL of the *E. coli* suspensions are −590.24 ± 16.84, −509.57 ± 23.19, and −181.58 ± 10.15 Hz/min under 15 V_pp_, respectively (see Figure 6). The respective values of the frequency shift rate for positive and negative control tests are −7.89 ± 0.47 and −7.46 ± 1.19 Hz/min. These results prove that utilizing DEP and gap method enhances fast and real-time detection of bacteria by collecting them at the gap of the V-shaped cantilever. Moreover, the positive control test shows that the sedimentation of bacteria on the V-shaped cantilever due to gravity and Brownian motion has a negligible impact on the real-time shift in the frequency. 

Repeated experiments (n = 3) for each concentration were performed. The final average frequency shift of each concentration is shown in Figure 6. The error bars indicate the standard deviation from the average frequency shift for each concentration. The graph shows that the average frequency shift at the end of DEP action for 10^5^ cells/mL is 1862.17 ± 439.79 Hz, for 10^4^ cells/mL is 1530.63 ± 395.93 Hz, and for 10^3^ cells/mL is 656 ± 86.87 Hz. These are the average of one point measurement from three experiments; their large standard deviations most likely come from the sensor variation from chip to chip and variations from testing samples. On the other side, frequency shift rate, calculated using all frequency shift data throughout the DEP action period for each experiment, demonstrated much higher consistency and smaller errors. In addition, the gap-method cantilever sensors have shown high repeatability in previously reported works [20,34]. 

**Figure 7:**
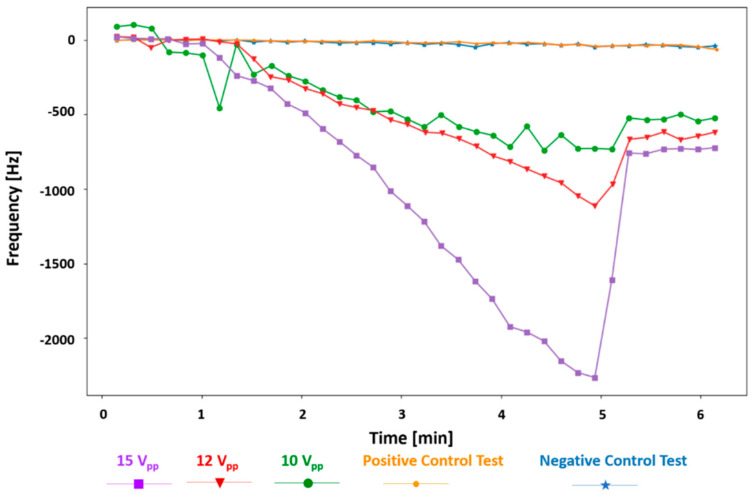
The frequency shift of the gap-method V-shaped microcantilever sensor for *E. coli* bacteria with a concentration of 10^5^ cells/mL when DEP signals with amplitudes of 15, 12, and 10 V_pp_ were applied and the positive and negative control tests.

The gap-method V-shaped microcantilever sensor was tested with different ac voltages applied for the DEP collection of the bacteria. As it is clear from Figure 7 and expected from Equation (3), higher voltages create a stronger DEP force; thus, more bacteria are collected at the gap, resulting in a higher frequency shift. The frequency shifts are −2264.3, −1113, and −727 Hz for applied voltages of 15, 12, and 10 V_pp_ in 10^5^ cells/mL *E. coli* suspensions, respectively. The frequency shift rate also increases by increasing the applied voltage. It is −590.24 ± 16.84 Hz/min for 15 V_pp_, −269.58 Hz/min for 12 V_pp_, and −167.46 Hz/min for 10 V_pp_. In addition, a lower voltage of 8 V_pp_ in the same concentration of *E. coli* was tested (not shown here). The frequency shift was −110.3 Hz after 4 min of the DEP being applied. This test was taken for 20 min (DEP on at minute 1 and off at minute 19) and showed a shift of −527.3 Hz at the end of the test right before the DEP signal was deactivated. The frequency shift rate for this test is −29.66 Hz/min. 

It has been shown previously that, in general, the gap-method microcantilever sensors have a higher sensitivity compared to the microcantilevers with conventional mass-change detection techniques. In addition, gap-method cantilevers show more than five times higher frequency shift compared to the frequency shift predicted by theory [20]. The reason for a rapid and strong response and a higher sensitivity of the gap-method sensors relies upon the fast collection of the particles to the free end of the cantilever, which is the most sensitive part of it. Moreover, the collected bacteria in the gap may bridge the free end of the vibrating cantilever to the fixed wall. This bridging adds higher damping to the cantilever vibration and results in higher frequency shifts compared with the conventional mass-change methods. However, the minimum sensitivity of the gap-method V-shaped sensor was calculated using the mass-change sensitivity in Equation (2). The effective mass of the V-shaped cantilever was calculated as 7155.7 µg, and the first-mode resonant frequency in water is 149.5 kHz, which gives a sensitivity of 95.73 ng/Hz. In addition, the limit of detection (LoD) of the sensor was calculated as 496.92 µg, with the same assumption. The LoD of the microcantilever biosensors is reported by dividing the effective cantilever mass by its quality factor (*m_n_*/Q) [17]. It is important to emphasize that the equations used to calculate the frequency shift (Equation (1)), the sensitivity (Equation (2)), and the LoD are considered under the mass change. In our sensor, the bacteria attracted to the gap area form bridges across the gap and increase damping; consequently, they amplify the frequency shift and further improve the sensitivity and LoD. In addition, the experimental sensitivity and LoD is also limited by the resolution of the frequency spectrum. Here, we use the first resonant mode of the cantilever for detection, and only the frequency range of 50–200 kHz is scanned, giving a frequency resolution of 31.25 Hz, which is high enough for the detection of bacteria even in much lower concentrations.

The gap-method V-shaped cantilever in this work introduces a longer gap, which provides a bigger interaction area to capture more particles. Even though in this paper we have focused on showing the fast detection ability of the V-shaped sensor, the longer gap extends the saturation time of the sensor. This means the sensor can perform sensing for a long time (more than one hour was tested, not shown here) before requiring any cleaning or reactivation. This property is an improvement over the previously reported gap-method microcantilever sensors, as they are saturated in a short time (around 6 min) [20,34]. It is also determined in the literature that the sensitivity of the cantilever sensors decreases when the added mass continues to increase, which the results show that it does not apply to this design [39]. This extended saturation time could potentially lead to the detection of biofilm maturation. The pDEP force might be an advantage to the biofilm maturation as well. Particles attracted to the high-intensity electric field areas chain up fast; thus, if enough bacteria are collected, then the biofilm might be created easily. Biofilms are created as a defense mechanism of the bacteria against the environment and monitoring their growth and studying their response to antibiotics are important, and some research in this area has been reported [40,41]. 

DEP force attracts all bioparticles to the gap area non-selectively. In this study, the sensing surface was not functionalized, which allows most of the attracted bacteria to be released when DEP is turned off. Surface functionalization with a selective antibody could be used to make the sensor specific to a target. A washing procedure will be needed to remove any temporally attracted non-target bacteria to reduce the noise, and no frequency shift recovery phenomenon is expected when DEP is turned off.

## 4. Conclusions

A V-shaped dynamic-mode microcantilever sensor was presented in this paper. The sensor works based on the gap method. There is a fixed structure at a 2 µm distance of the free end of the microcantilever. Two independent ac electric potentials were applied to drive the microcantilever and attract bacteria to the gap by DEP force. Samples of *E. coli* bacteria suspended in distilled water were tested in 10^5^–10^3^ cells/mL concentrations, and respective frequency shifts of −2264.3, −1929.3, and −755 Hz for each concentration were detected in 4 min. Additionally, the results showed that, by increasing the amplitude of the applied electric potential, higher frequency shifts were observed, as expected. For now, the authors only tested the sensor chip for *E. coli* concentrations up to 1000 cells/mL. However, it can be reduced to lower bacteria concentrations by a slight change in the test setup, for example, using flowing flow samples instead of stagnant samples.

## Data Availability

Not applicable.
